# Review of backscatter measurement in kilovoltage radiotherapy using novel detectors and reduction from lack of underlying scattering material

**DOI:** 10.1120/jacmp.v14i6.4358

**Published:** 2013-11-08

**Authors:** David J. Eaton, Paul J. Doolan

**Affiliations:** ^1^ Radiotherapy Physics Royal Free Hospital London UK

**Keywords:** backscatter factor, GAFCHROMIC film, kilovoltage, radiotherapy, lead shielding

## Abstract

Lack of underlying material can lead to dose reduction in kilovoltage radiotherapy treatments because of backscatter reduction. Conversely, the use of lead shielding can lead to large dose enhancement close to the lead interface. GAFCHROMIC film has been shown to be of use in verification of local backscatter factors compared to reference data in codes of practice, but careful handling and multiple readings are required to reduce systematic uncertainties to between 3% and 4%. Monte Carlo modeling of the specific treatment unit should be performed in cases which are found to differ from reference values before alternative values are adopted clinically, but these cases are expected to be few. GAFCHROMIC film may also be used to estimate backscatter reduction more readily than customized ionization chambers, for a range of beam qualities, applicator sizes and depth, with and without lead shielding. Differences were found between different studies, and it is not clear to what extent these are due to variation in equipment and/or technique. However, a layer of wax around lead shielding of 1 mm thickness should be sufficient to eliminate lead enhancement effects for all kilovoltage energies from 40 kV to 300 kV

PACS numbers: 87.55.Qr, 87.56.jk

## I. INTRODUCTION

Superficial tumors are commonly treated using kilovoltage X‐ray units with tube potentials in the region of 40–300 kV. Calibration is typically performed using air ionization chambers, and converted to dose in water using the backscatter factor (BSF), defined as the ratio between the dose at the surface of a full scatter water phantom and the dose with no phantom. Measurement of these values has been challenging because of detector size and energy response. Therefore, dosimetric codes of practice contain data for a range of beam qualities, source‐to‐surface distances (SSD), and field sizes, which may be used by a center following spot checks against their own equipment. These data are based on Monte Carlo simulations verified by measurements with a custom parallel plate ionization chamber.^(^ ^1^ ^,^ ^2^ ^)^ More recently, GAFCHROMIC EBT film^(^ ^3^ ^,^ ^4^ ^)^ and its replacement EBT2^(56)^ (International Specialty Products, Wayne, NJ) have been used for local verification of BSF, but with varying degrees of success. Radiochromic film has the advantages of small thickness, ease of handling, and tissue equivalence, although EBT2 may have greater energy dependence, batch variation, and nonuniformity.

In clinical conditions, the depth of underlying material may be limited by lack of tissue (for example, treatment of keloids on the pinna of the ear^(7)^) and BSF may be greatly reduced in these circumstances. Lead shielding is sometimes used behind the target volume, which can lead to both dose reduction from lack of underlying tissue and dose enhancement at short distances because of backscatter from the lead. Therefore, lead shields are typically covered by a layer of wax or plastic film to absorb this additional scatter. Quantification of these effects has been performed using ionization chambers,^(^ ^8^ ^,^ ^9^ ^,^ ^10^ ^,^ ^11^ ^,^ ^12^ ^)^ along with Monte Carlo simulation.^(13)^ Studies have also been performed to investigate the effect of bone interfaces^(^ ^14^ ^,^ ^15^ ^)^ The aims of this paper are twofold: (i) to review the available evidence for the use of GAFCHROMIC film and other novel detectors for the measurement of BSF; and (ii) to investigate the use of EBT2 film in measurements of backscatter reduction, which has not been done before for reduced depths and in the presence of lead shielding. Particular attention will be given to very small depths of scattering material (< 5 mm) which has not been explored by most other authors. Different reported datasets will be compared to establish the usefulness of EBT2 film in BSF measurement and to review differences in the amount of reduction found with different systems and methods.

### A. Background

#### A.1 Backscatter factors

Calibration of low kilovoltage energies is typically performed in air, using an air‐kerma calibration method. Both the AAPM^(1)^ and IPEM^(2)^ codes of practice describe this method for energies of 40–300 kV and 50–160 kV (later extended to 300 kV^(16)^), respectively. The in‐air method is preferred over the alternative in‐phantom calibration at energies greater than 100 kV as it gives a more accurate dose at the surface, which is the usual prescription point. It is also quick to set up and requires less equipment. The formalism gives absorbed dose to water at the surface of a full scatter water phantom:

(1) $$D_{w,z=0}=M_{air}N_{K}B_{w}{\left[{\left(\frac{{\bar{\mu }}_{en}}{\rho }\right)}_{w/\text{air}}\right]}_{\text{air}}$$

where $M_{\text{air}}$ is the chamber reading in air corrected to standard temperature and pressure, $N_{K}$ is the air kerma calibration factor for the chamber at the given beam quality, $B_{w}$ is the backscatter factor (defined as the ratio of water collision kerma at the surface of a full scatter water phantom to the same point on the beam axis with no phantom present), and ${\left[{\left({\overset{―}{\mu }}_{\text{en}}/\rho \right)}_{w/\text{air}}\right]}_{\text{air}}$ is the mass energy absorption coefficient ratio of water to air, averaged over the photon spectrum in air. The AAPM code also includes $P_{\text{stem},\text{air}}$, which is the chamber stem correction factor and can be taken as unity if the same field size is used for standards laboratory calibration and local measurement.

Backscatter factors are tabulated in these two codes with respect to beam quality (defined by half value layer, HVL), field size and SSD. The tabulated values are based on Monte Carlo calculations verified by measurement with a custom‐built thin parallel plate chamber, and have been used as the gold standard for commissioning of new treatment units for over a decade. Local measurement of these factors is confounded by the finite size of many common detectors, leading to perturbation and partial volume effects, as well as the potential lack of water‐equivalence of detectors and solid phantom materials at kilovoltage energies. Recently, a number of novel detectors have been investigated to see if they can be used for local verification, including GAFCHROMIC film,^(^ ^3^ ^,^ ^4^ ^,^ ^5^ ^,^ ^6^ ^)^ thermoluminescent dosimeters (TLDs),^(17)^ and optically stimulated luminescent dosimeters (OSLDs).^(6)^ Results of these studies are discussed below, alongside data from this current work.

It has been suggested that the use of generic (code of practice) values for BSF increases the uncertainty in absorbed dose calculations^(^ ^18^ ^,^ ^19^ ^)^ although only for one of the two clinical beams studied by Munck af Rosenschöld et al.^(18)^ Chica et al.^(19)^ established a linear relationship of dose with respect to homogeneity coefficient and tube voltage, and suggested HVL and tube voltage together could be used to more fully characterize X‐ray beams. They later extended this concept to backscatter factors^(20)^ and derived an empirical formula for the calculation of BSF based on HVL and tube voltage, but only for an impractical SSD of 100 cm. Currently, codes of practice values are only defined in terms of HVL, although the AAPM code recommends that the chamber should be calibrated at beam qualities sufficiently close to the user's beam in terms of both HVL and tube potential to minimize uncertainties in this approach. Total uncertainty for the in‐air method is reported as 3.5% (1 o), including 1.5% for backscatter factor and 2% for beam quality differences.^(1)^

#### A.2 Backscatter reduction

Reduction of backscatter with lack of underlying material was thoroughly investigated by Klevenhagen^(8)^ using a custom‐build parallel plate ionization chamber and a titanium‐doped polystyrene phantom. He found that the relative reduction was greater for larger field sizes, higher beam qualities, and longer SSDs. Therefore, the slope of the backscatter reduction curve is related to the slope of a depth‐dose curve in full scatter conditions. As the range of the scatter increases, giving an increased percentage depth‐dose value at a given depth in full scatter conditions, so does the amount of material required to approximate full scatter. Hence, the amount of backscatter with a given small thickness of underlying scattering material is reduced. A multiple exponential fit was made to these data to allow prediction for a given thickness of material, beam quality, SSD, and field size.

Other authors extended this experimental approach to the reduction in underlying scattering material due to the presence of a lead interface, which is typically used for shielding behind the treatment area.^(^ ^9^ ^,^ ^10^ ^)^ They found a similar dependence of the relative backscatter reduction on increasing beam quality (up to 8 mm Al) and increasing field size, but less reduction with the lead interface compared to the data for an air interface given by Klevenhagen.^(8)^ This suggested some scatter contribution from the lead even at larger depths, although Healy et al.^(12)^ later found the influence of the lead to be minimal for thicknesses of material before the lead greater than 3 cm. It was concluded that the backscatter reduction was mainly because of the lack of scatter from water rather than the effect of the lead. However, the finite size of the detectors used in these two studies restricted the minimum depth to 3 mm or 6 mm, respectively, so any enhancement close to the interface was not seen. Das and Chopra^(11)^ measured the changes in backscatter with a range of parameters, including distances very close to the interface, and found dose enhancement could be 5–15 times greater at the surface, but this was reduced to below unity (i.e., dose reduction) within the first half‐millimeter. The enhancement reached a peak at a beam quality of 1.0 mm Cu (approximately 12 mm Al^(2)^), owing to the decreasing photoelectric cross section at higher energies. This was found by other authors as well, such as Huq et al.^(9)^ who found less perturbation (in their case, less reduction) at 250 kV (0.5 mm Cu, or 8 mm Al) compared to 100 kV (3.5 mm Al). However, the beam quality of the largest reduction at depth is different to the largest surface enhancement found by Das and Chopra,^(11)^ and may be more machine dependent, as will be discussed further below.

Hill et al.^(13)^ performed Monte Carlo simulation of backscatter reduction with lead shielding, but only for depths of 5 mm or greater, and found the largest reduction for the largest field sizes, as reported by other authors described above. They also found less reduction for a 135 kV beam (7.2 mm Al) compared to a 100 kV beam (4.0 mm Al), similar to the results of Huq et al.^(9)^ Good agreement was found with measured values, but a systematically larger reduction than found by Lanzon and Sorell,^(10)^ which was ascribed to differences in machine design and SSD. The beam spectra varied little from the full scatter conditions, reinforcing the conclusion that reduction at these depths is caused mainly by lack of scatter from water rather than perturbation by the lead.

## II. MATERIALS AND METHODS

Measurements were made using a Gulmay D3225 superficial therapy x‐ray unit (Gulmay Medical, Camberley, UK) with beam qualities listed in Table 1. Circular open‐ended applicators with an SSD of 20 cm were used for the lower energies (60–160 kV) and square closed ended applicators with an SSD of 50 cm were used for the highest energy (220 kV). The reference applicator size of 10 cm was used for all energies, and a 3 cm diameter applicator was also used for the lower energies. Calibration of the unit was performed using cylindrical ionization chambers in air for all energies, according to the IPEM code of practice and its addendum.^(^ ^2^ ^,^ ^16^ ^)^

**Figure 1:**
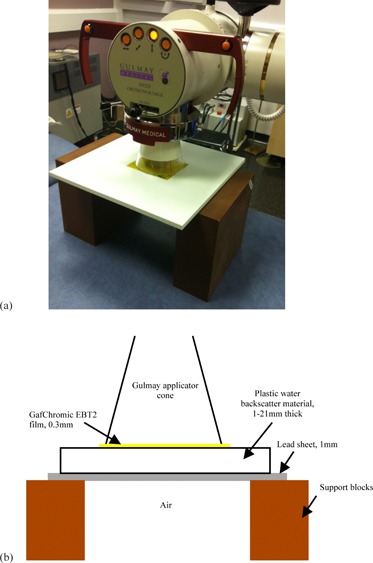
Photograph (a) of a film piece positioned on top of a slab of PWDT to simulate reduced backscatter conditions, and diagram (b) of setup with a lead sheet beneath the solid water slab.

**Figure 2:**
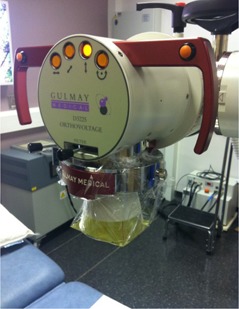
Film piece positioned with cling film at the end of the applicator, simulating conditions of no backscatter.

GAFCHROMIC EBT2 film has an asymmetrical construction with a thickness of 0.3 mm and the active layer at a physical depth of 0.1 mm. This depth is sufficient to absorb contaminant electrons produced by the collimation. All films were taken from a single batch, with each film cut into several smaller pieces. Their orientations and positions with respect to the original sheet were tracked throughout the study. In preparation for irradiation, films were placed on top of blocks of solid water material at the end of the applicator. A 10 cm thick block of WT1 solid water material (St. Bartholomew's Hospital, London, UK) was used for full backscatter conditions. For reduced backscatter conditions (backscatter thickness ranging from 1 mm to 21 mm), sheets of Plastic Water Diagnostic Therapy (PWDT, CIRS, Norfolk, VA) or Plastic Water Low Range (PWLR, CIRS) were supported with an air gap beneath, as shown in Fig. 1. Measurements with lead were performed with a 1 mm thick sheet underneath the solid water. Conditions of no backscatter were simulated by attaching the film to the end of the applicator with plastic wrap (cling film), sufficiently far from any other scattering material, as shown in Fig. 2. A dose of 2 Gy was used for the 20 cm SSD applicators (60–160 kV, 300 MU/min), and 0.5 Gy for the 50 cm SSD applicator (220 kV, 50 MU/min) because of the lower dose rate.

Calibration of the film was performed using full scatter conditions and irradiation of film pieces to known doses over the expected range of backscatter factor values. EBT2 (and EBT) film are nonlinear with respect to dose, so it is important to acquire calibration films over the full range of subsequently measured values, and at sufficiently frequent dose intervals, to allow accurate fit of the response. Films were handled with gloves to reduce fingerprint contamination, and processed following the method of Kairn et al.^(21)^ to correct for nonuniformities in the film and scanner response. Each sheet was scanned before and 72 hours after irradiation in consistent orientation on an Epson Perfection V700 flatbed scanner (Epson, Long Beach, CA). After 2–3 warm‐up scans, films were read out using the following settings: positive film (transmission, with area guide), 75 dpi, 48 bit RGB color, no corrections. The red channel was extracted using ImageJ (Wayne Rasband, National Institutes of Health, http://rsb.info.nih.gov/ij/), and the net optical density for each pixel (i,j in Eq. (2)), netOD, was calculated using:

(2) $$\text{netOD}=\text{log}\left(\frac{P_{red,pre}(i,j)}{P_{red,post}(i,j)}\right)$$

where $P_{\text{red},\text{pre}}(i,\;j)$ is the pixel value in the pre‐irradiation red channel image and $P_{\text{red},\text{post}}(i,\;j)$ is the pixel value in the postirradiation red channel image. The measured reading was sampled using a circular region of interest, 30 pixels in diameter (1.0 cm). Optical density values were then converted to dose by linear interpolation into the calibration curves. BSF was calculated as the ratio between full scatter and no scatter conditions. Reduced backscatter was expressed as the ratio of full scatter conditions following the approach of Lanzon and Sorell^(10)^ to give the backscatter reduction factor (BSRF):

**Table 1:** X‐ray beam parameters used for measurements

*Tube Potential*	*Beam Current*		
*(kV)*	*(mA)*	*Inherent Filtration*	*Half‐value Layer*
60	14.7	1.0 mm Al	1.3 mm Al
120	10.2	0.5mmAl+0.10mmCu	4.9 mm Al
160	7.7	1.5mmAl+0.15mmCu	8.0 mm Al (0.5 mm Cu)
220	12.8	1.0mmAl+0.25mmCu+0.45mmSn	2.2 mm Cu

(3) $$\text{BSF}=\frac{D(\text{full}\;\text{scatter})}{D(\text{no}\;\text{scatter})}$$

(4) $$\text{BSRF}=\frac{D(\text{reduced}\;\text{scatter})}{D(\text{full}\;\text{scatter})}$$

## III. RESULTS

Measured BSFs are listed in Table 2, with agreement within 2% compared to IPEM code of practice values.^(^ ^2^ ^,^ ^16^ ^)^ Scan time after irradiation was found to have little effect on readings, so long as calibration films were scanned after a consistent delay. Reproducibility between different sessions was found to be within 3%, although a few readings were discarded that were much lower than repeated values. This was thought to be due to bowing of the film away from the surface of the solid water. Also, for later repeat measurements, the film pieces were placed only in the central region of the scanner, to reduce the dose dependent nonuniformity of scanner response towards the edges of the plate. Estimates of the total measurement uncertainty are given in Table 3. These data are based on the observed local variation, for example in machine output, nonuniformity of film and scanner, and pixel values over the measurement region, and the estimates of other authors.^(^ ^4^ ^,^ ^5^ ^,^ ^22^ ^,^ ^23^

Measured BSRF values with respect to thickness of material are shown in 3, 4, for 60 kV and 120 kV beams and applicator sizes of 3 cm and 10 cm diameter. On the same graphs, predicted values from two other authors have been plotted for comparison. The empirical formulae suggested by Klevenhagen^(8)^ were used to generate values for the same beam quality, SSD, and field sizes as the local conditions, and converted from fraction of full backscatter to BSRF using BSFs in the IPEM code of practice.^(2)^ The formula suggested by Lanzon and Sorell^(10)^ for BSRFs with underlying lead was used to calculate values using parameters for the closest reported beam qualities of 1.4 mm Al and 4.7 mm Al (compared to 1.3 mm and 4.9 mm Al, respectively, used in this study), and an SSD of 10 cm. These parameters were based on measured data for depths of 6–70 mm and field sizes of 15–79 mm, so values have not been extrapolated beyond this range.

Figure 5 shows BSRF with respect to beam quality for a field size of 10 cm, with no backscatter material at all (in air), no additional material between the film and the lead sheet, and 1 mm solid water between the film and lead. The thickness of the polyester substrate before the active layer in the film is 0.097 mm, so data from Das and Chopra^(11)^ for distances to interface of 0.1 mm and 1.1 mm and an applicator size of 10cm×10cm are also plotted for comparison. Beam qualities quoted in mm Cu were converted to mm Al to give a continuous plot using data from the IPEM code of practice. Figure 6 shows BSRF with respect to beam quality for 10 mm depth of backscatter material before the lead shielding, for both applicator sizes measured locally. Values from three other authors^(^ ^11^ ^,^ ^12^ ^,^ ^13^ ^)^ for various field sizes are also plotted for comparison.

**Table 2:** Backscatter factors (BSF) measured locally compared to IPEM code of practice data.^(^ ^2^ ^,^ ^16^ ^)^

*Tube Potential (kV)*	*Applicator Size (cm)*	*Measured BSF*	*IPEM BSF*	*Difference (%)*
60	3.0	1.132	1.114	+1.6
	10.0	1.185	1.179	+0.5
120	3.0	1.143	1.164	−1.8
	10.0	1.309	1.335	−1.9
160	10.0	1.369	1.350	+1.4
220	10.0	1.296	1.292	+0.3

**Table 3:** Estimate of BSF measurement uncertainties using EBT2 film

*Component*	*Uncertainty (%)*
Output stability	0.5
Film positioning	1.0
Film nonuniformity	1.5
OD variation over pixels	1.5
Scanner variation	1.5
Film calibration	1.0
Water equivalence of phantom	1.0
Total	3.2

**Figure 3:**
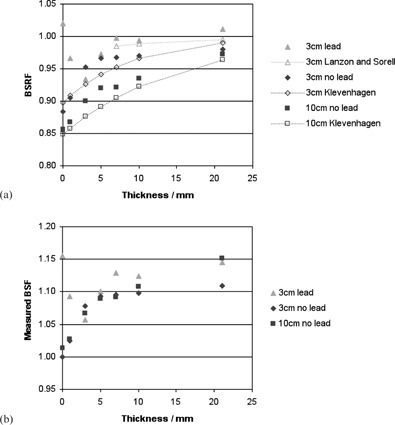
BSRF values (a) measured locally, and predicted by two other authors, for beam qualities of approximately 1 mm Al and applicator sizes of 3 cm and 10 cm; and locally measured values (b) expressed as BSF. Error bars have been omitted for clarity.

**Figure 4:**
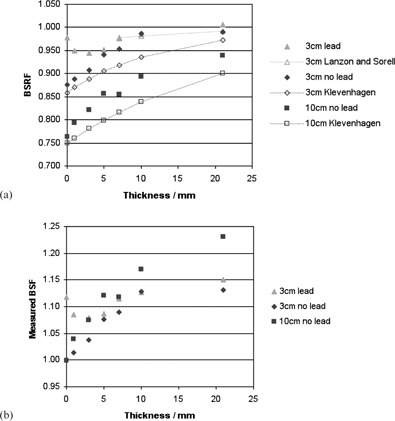
BSRF values (a) measured locally, and predicted by two other authors, for beam qualities of approximately 5 mm Al and applicator sizes of 3 cm and 10 cm; and locally measured values (b) expressed as BSF. Error bars have been omitted for clarity.

**Figure 5:**
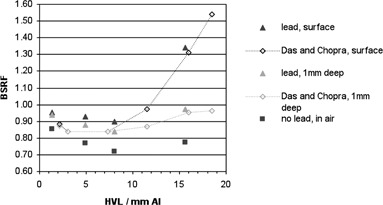
BSRFs measured locally and in one other study, for applicator size of 10 cm, at the surface of the lead sheet, with 1 mm depth of solid water before the lead and in‐air with no lead. Error bars have been omitted for clarity.

**Figure 6:**
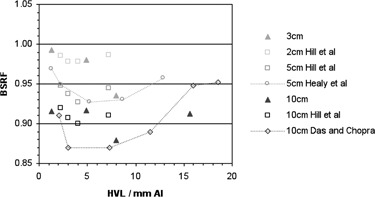
BSRFs measured locally and in three other studies, for 10 mm depth to lead and a range of field sizes. Error bars have been omitted for clarity.

## IV. DISCUSSION

### A. Backscatter factors

Accurate measurement of BSFs relies on using a dosimeter of minimal thickness or a method to extrapolate to zero thickness, as used by contributors to the code of practice datasets. Butson et al.^(3)^ first investigated the use of GAFCHROMIC film in this respect, and found good agreement with IPEM data, as shown in Table 4 alongside results from this study and other authors described below. Butson and colleagues also used measurements with a stack of multiple films to extrapolate back to zero thickness and found the difference between these values and those acquired with a single layer of film to be within 2%. Therefore, although the film had a finite thickness, single pieces could be used to verify local BSFs to reasonable accuracy. Kim et al.^(4)^ also measured BSFs with EBT film and found good agreement. They simulated the treatment unit using an updated Monte Carlo code and found BSFs agreed with AAPM data within 1%, concluding that these data were still valid (at least for their unit) and that EBT film could be used to measure BSFs locally over the full energy range (50–280 kV). Smith et al.^(5)^ used GAFCHROMIC EBT2 film to measure BSFs on the same treatment unit as in the Kim study. They found agreement with AAPM data within measurement uncertainties, as long as the film was used in a consistent orientation. Measured uncertainties were higher than for EBT film, because of increased inhomogeneity in the EBT2 sensitivity; therefore, it was recommended that multiple readings be taken. They also suggested that the film should be left 96 hours to stabilize, but this was the only time delay used in the study, so does not exclude other times as long as the same interval is used for the calibration and measurement films. Results from our center gave similar levels of agreement and uncertainty to these authors, and support their conclusions on the usefulness of GAFCHROMIC film as a dosimeter for BSF measurement.

**Table 4:** Summary of reported BSF measurements using novel detectors

*Study*	*Machine*	*Beam Quality (HVL)*	*Detector*	*Reference Data*	*Maximum Difference to Reference Data*	*Measurement Uncertainty*
${\text{Butson and Cheung}}^{\left(3\right)}$	Gulmay D3300	50–150 kV (1.4mmAl−0.6mmCu)	GAFCHROMIC EBT	IPEM	1.8%	Not given
${\text{Kim et al}.}^{\left(4\right)}$	Pantak DXT300	50,100,280 kV (1.5mmAl−3.2mmCu)	GAFCHROMIC EBT	AAPM	2%	2.5%
${\text{Smith et al}.}^{\left(5\right)}$	Pantak DXT300	50,100,280 kV (1.5mmAl−3.2mmCu)	GAFCHROMIC EBT2	AAPM	3.5%	4.0%
${\text{Nelson and Hill}}^{\left(17\right)}$	Pantak Therapax (DXT) 300	75–300 kV (2.2mmAl−3.2mmCu)	LiF TLD	AAPM & IPEM	~6%	1.1%−4.7%
${\text{Mart et al}.}^{\left(6\right)}$	Gulmay D3100	20, 100 kV (0.08, 4.0 mm Al)	GAFCHROMIC EBT2	AAPM^a^ IPEM	11%, 7%	2%−7%
			OSLD	AAPM^a^ IPEM	14%	1%−10%
This study (2013)	Gulmay D3225	60–220 kV (1.3mmAl−2.2mmCu)	GAFCHROMIC EBT2	IPEM	1.9%	3.2%

a Originally, data for 20 kV were compared to a very old publication (BJR supplement 10, 1961) which has since been updated three times and currently (BJR supplement 25, 1996) agrees with AAPM code of practice data within 1%. Therefore current AAPM code of practice values are used for this comparison.

Nelson and Hill^(17)^ used two types of TLDs to measure BSFs and also performed Monte Carlo simulation of the specific beams used. They found moderate agreement, with measurements at all but one energy within 5% of AAPM and IPEM code of practice data and Monte Carlo results. They suggested that TLDs can be used to measure BSFs in cases where data are not available, but for their clinical system, IPEM data was valid. Mart et al.^(6)^ used both EBT2 film and OSLDs to measure BSFs for two energies, including one with a very low beam quality (<40kV). They found differences of greater than 10% both between measured values with the two dosimeters and between measured values and reference data. Measurement uncertainties were also much larger than other studies, up to 7% for EBT2 film and up to 10% for OSLDs. Systematic differences between measured and reference datasets were ascribed to spectral differences between the local and historical beam qualities. However, differences were often within quoted measurement uncertainties, and other authors^(^ ^3^ ^,^ ^4^ ^)^ found good agreement with reference data using a 100 kV beam of similar HVL (3.7 and 3.8 mm Al compared to 4.0mm Al). In addition, datasets for both detectors and beam energies contain anomalies such as a lack of continuous increase of BSF with field size, which would be expected.

Munck af Rosenschöld et al.^(18)^ performed Monte Carlo simulation of two energies on a Gulmay D3300 unit, and found differences of up to 3% compared to AAPM values. However, absorbed dose calculations for different in‐air codes of practice agreed within 1% for all energies (30–200 kV) and overall estimated uncertainties were between 3% and 4%, similar to the codes of practice. Chica et al.^(19)^ modeled 74 hypothetical beam spectra with a range of HVLs and tube voltages, and found uncertainties of between 2% and 4% in BSFs for 20–150 kV beams. These data were not compared to code of practice values, however, and uncertainties in dose were also between 2% and 4%. Chica and colleagues extended this approach to derive an empirical formula for BSFs based on simulation of the hypothetical beams, but only for an impractical SSD of 100 cm, which is much longer than used typically in clinical practice and, again, the data were not compared to code of practice values. BSFs were measured for 20 experimental beams and agreed with formula predicted values within 5%. Measurements used a Farmer‐type ionization chamber, but perturbation effects are not discussed, so this should be interpreted as a verification of the measurement technique by the model, rather than the other way round, as suggested. Therefore, the chamber accuracy is slightly poorer than film methods described above. The measured beams also used a wide range of filtration and tube voltage to give the same HVL, so may not be representative of typical clinical settings.

Several authors^(^ ^3^ ^,^ ^6^ ^,^ ^17^ ^)^ have ascribed the differences between measured and code of practice values to differences in spectral distribution of local beams. However, in all of these cases, the differences are of the same order as the measurement uncertainties, so a more reasonable conclusion would be that the local factors agree with reference data within inherent uncertainties.

Recently, a new brand of GAFCHROMIC film has become available, EBT3 (International Specialty Products), which could also be used for BSF measurement. The formulation of this film is very similar to EBT2, but with a symmetrical construction to reduce orientation uncertainties and with silica particles in the coating to reduce Newton's ring artifacts.^(24)^ Therefore, it is expected to give similar performance to EBT2 results given in this work, although there may be greater issues with delamination when cutting the film into the small pieces used in these studies.

Therefore, it can be concluded that, while there is some evidence of the usefulness of GAFCHROMIC film for verification of local BSFs, there is little evidence that established code of practice values will not be applicable to clinical units. Other detectors currently have measurement uncertainties too great to be useful for verification, and even GAFCHROMIC film must be handled very carefully to reduce uncertainties to the 3%‐4% level needed for verification. In this study, good agreement was found with IPEM values, but repeat readings were frequently necessary to reduce variation between measurements and identify systematic outliers caused by air gaps when positioning the film relative to the backscatter material or nonuniformity across the scanner plate. Only if Monte Carlo simulation of specific units and film measurements are found to be in agreement and significantly different to reference code of practice values should local BSFs be considered. However, given the low number of manufacturers of this equipment and simulation studies performed on current units described above, this is unlikely in the near future.

### B. Backscatter reduction

Backscatter reduction with lack of underlying material is greatest for larger field sizes and smaller depths of material, as found by measurements from our center and all other investigators highlighted previously. Since the variation with field size has been shown to be smooth and continuous by other authors, only two representative sizes were measured locally, in favor of more depths and beam energies. Values in this study are systematically higher than predicted by Klevenhagen,^(8)^ except for the lowest beam qualities, depths, and field size. Healy et al.^(12)^ used a cylindrical ionization chamber to measure backscatter reduction with and without lead for a number of low energies (1.3–13 mm Al) and small field sizes. They also found a 2% increase in BSRF values compared to those predicted by Klevenhagen's formulae, which was attributed to differences in measurement technique and X‐ray unit — although this could instead be attributed to measurement uncertainties. Also, they only measured depths of 5 mm and larger, so any crossover at the smallest depths would not have been observed.

Recently, influence of backscatter material and thickness has been modeled for diagnostic beams also, showing reductions of up to 12% for the highest energy, largest field size, and 5 cm thick phantoms compared to 15 cm full scatter conditions.^(25)^ Correction factors for the use of PMMA instead of water were 3%‐10%. Appreciation of the magnitude of these effects is important in both disciplines, and should be mitigated or accounted for clinically, as appropriate.

Effects of bone interfaces have been investigated by two recent studies. Butson et al.^(14)^ used a parallel plate chamber to measure reduction of dose with a bone tissue interface close to the measurement surface, with larger reductions measured for larger field sizes. They also found an enhancement effect very close to the interface similar to lead, though much smaller and not dosimetrically significant. The largest reduction was found using a 100 kV beam (3.7 mm Al), with less reduction at 150 kV (0.6 mm Cu, or 10 mm Al). They suggest that GAFCHROMIC film would be a suitable dosimeter to measure these effects *in vivo*. Chow and Owrangi^(15)^ performed Monte Carlo simulation of a single 105 kV clinical beam and found surface dose reduction up to 8% for water depths of 0.5–5 mm between the surface and the bone interface, along with larger reductions for larger field sizes. They did not, however, extend these simulations to very shallow depths, so did not observe any enhancement. It is unfortunate that the few Monte Carlo studies into backscatter perturbation at high density interfaces^(^ ^13^ ^,^ ^15^ ^)^ have not simulated the smallest distances, which are the very conditions most difficult to measure experimentally. Further work in this area of enhancement at shallow depths is warranted.

When lead is positioned behind the backscattering material, the BSRF values are typically increased, and at very small depths the behavior changes from dose reduction to dose enhancement. Many previous studies of backscatter reduction with lead only measured at distances greater than 5 mm from the lead surface^(^ ^10^ ^,^ ^12^ ^,^ ^13^ ^,^ ^26^ and so did not observe this effect. Healy et al.^(12)^ did show some evidence of an uplift at the lowest measured depth and beam energies, but this was attributed to a possible perturbation effect in the chamber. However Das and Chopra^(11)^ showed in detail the crossover region with measurements right up to the surface of the lead, and their results are thus also included in Fig. 5. At the surface, enhancement can be many times the value without lead, but this decreases rapidly in the first 0.1 mm to the values shown. The finite thickness of the film results in measurements that are a small effective distance from the surface, but the enhancement effect can still be clearly seen, and this setup is more practical than ionization chamber methods. Good agreement was found with values proposed by Lanzon and Sorell,^(10)^ but the exponential form of their proposed formula does not account for the dose enhancement at short distances, so the use of this empirical fit will be limited.

In clinical practice, lead shielding is covered with either plastic wrap (cling film) or wax to mitigate the effects of dose enhancement. Mayles et al.^(27)^ suggest the use of 1 mm wax for lead cutouts at energies up to 140 kV, but provide no source data. However, results from this study suggest that this thickness of wax is sufficient for higher energies as well, in agreement with Das and Chopra^(11)^ who suggest that 1 mm of tissue‐equivalent material may be used up to 300 kV.

Figure 6 shows the variation with HVL for measurements from our center and three other studies, for a single depth of 10 mm. Healy et al.^(12)^ report differences to Lanzon and Sorell^(10)^ of 2.5% for a beam quality of 2 mm Al, but agreement with Hill et al.^(13)^ within 1%. However, it can be seen from Fig. 6 that the variation is much greater across a range of beam qualities. In fact, there is little agreement between the four studies in terms of the BSRF for larger field sizes and the position of the minimum in the curves. Values ranging between 4 mm Al and 12 mm Al have been reported^(^ ^9^ ^,^ ^11^ ^,^ ^12^ ^,^ ^13^ ^,^ ^14^ ^)^ for different depths, compared to a value from our center of approximately 8 mm Al, as shown in 5, 6. This variation may be because of different equipment or methods used by different authors; however, there is currently insufficient evidence for this and further investigation by Monte Carlo simulation will be necessary to identify the causes. Until that time, however, GAFCHROMIC film is a practical and readily available tool that may be used by local centers on their own equipment to appreciate and quantify these effects, with the caveat of potential uncertainties, as discussed for BSF measurements.

## V. CONCLUSIONS

Reduction of backscatter with lack of underlying material and enhancement close to lead shielding are effects that should be considered when planning kilovoltage treatments. GAFCHROMIC film offers a practical and readily available method for verification of backscatter factors and estimation of perturbation effects in a local center, although careful handling and repeated measurements are needed to reduce uncertainties to between 3% and 4%.

For backscatter factors (conditions of full scatter), variation between clinical equipment has not been sufficiently proven and it is expected that code of practice values can continue to be used for the majority of situations. Local values should only be introduced following specific Monte Carlo modeling of the beams involved and verification by measurement, but these situations are expected to be rare.

For backscatter reduction, considerable variation has been found between different equipment; therefore, centers are advised to quantify effects for their own equipment unless specifically reported elsewhere. Further modeling of perturbation effects at short distances would be useful, though enhancement from lead shielding can be mitigated by 1 mm wax for energies from 40–300 kV.
